# Tanshinone IIA inhibits cell viability and promotes PUMA-mediated apoptosis of oral squamous cell carcinoma

**DOI:** 10.7150/jca.84537

**Published:** 2023-08-06

**Authors:** Shuangze Han, Xinfang Yu, Ruirui Wang, Xiaocong Wang, LuLu Liu, Qing Zhao, RongBo Xie, Ming Li, Zhong Su Zhou

**Affiliations:** 1 The Third Hospital of Changsha, Changsha 410015 Hunan, People's Republic of China.; 2 Department of Ultrasound, Union Hospital, Tongji Medical College, Huazhong University of Science and Technology, Wuhan 430022, China.; 3 Department of Medicine, Baylor College of Medicine, Houston, TX, 77054, USA.; 4 Department of Radiology, the Third Xiangya Hospital, Changsha, 410013, China.; 5 Hunan University of Chinese Medicine Affiliated Stomatological Hospital, Changsha 410208 Hunan, People's Republic of China.; 6 Changsha Stomatological Hospital, Changsha 410004 Hunan, People's Republic of China.

**Keywords:** Tanshinone IIA, PUMA, oral squamous cell carcinoma

## Abstract

Apoptosis alteration is responsible for tumorigenesis and tumor resistance to therapies. The natural product Tanshinone IIA (Tan IIA) exhibits potent inhibitory effects against various tumors. However, the effect of Tan IIA on apoptosis and its underlying mechanism remains elusive in oral squamous cell carcinoma (OSCC). Here, we demonstrated that Tan IIA dose-dependently suppressed cell viability and colony formation in CAL27, SCC4, and SCC25 cells. Moreover, Tan IIA inhibited Akt activation from inducing Foxo3a dephosphorylation and PUMA-mediated apoptosis. PUMA or Foxo3a knockdown compromised the inhibitory effect of Tan IIA on OSCC cells. Tan IIA administration inhibited CAL27-deprived xenograft tumor growth and increased PUMA expression in vivo. Tan IIA synergistically intensified the efficacy of CDDP/5-FU-based chemotherapy on OSCC cells. Overall, our results revealed that Tan IIA exerted potent antitumor effects via promoting PUMA-mediated apoptosis in OSCC cells.

## Introduction

Oral squamous cell carcinoma (OSCC) is the most common human oral malignancy, accounting for approximately 90% of all oral cancer cases^1^, ^2^. The incidence and mortality have increased over the past decades, with an overall 5-year survival rate for OSCC ranging from 50% to 60%^2^-^4^. Currently, the therapeutic criterion for OSCC includes surgery, regional radiotherapy, and systemic chemotherapy^5^. However, approximately 50% of OSCC patients have poor prognosis^1^, ^6^. Thus, OSCC prevention and treatment must elucidate underlying mechanisms and identify novel antitumor agents.

Tanshinone IIA (Tan IIA) is a major functional compound extracted from Danshen (*Salvia miltiorrhiza Bunge*), demonstrating a wide range of anticancer effects^6^, ^7^. Increasing evidence delineated that Tan IIA exhibited anti-oxidant and anti-inflammatory properties^8^, ^9^. Tan IIA promoted autophagy-induced cell death in diverse forms of cancer cells^6^, ^10^. Administration of Tan IIA targeted Aurora B kinase to suppress tumor growth and overcome radioresistance in OSCC cells 11. Tan IIA suppressed HK2-mediated aerobic glycolysis in OSCC cells^2^, suggesting that Tan IIA is a promising antitumor agent against human cancers.

In this study, we determined the inhibitory effect of Tan IIA on OSCC cells and further unveiled the novel underlying mechanism of Tan IIA administration against OSCC.

## Materials and methods

*Cell culture and agents.* CAL27, SCC4, and SCC25 cells were purchased from American Type Culture Collection (ATCC, Manassas, VA). All cells were cultured in DMEM medium supplemented with 10% Fetal Bovine Serum (FBS) and 1% penicillin-streptomycin at 37°C with 5% CO_2_. All cells were subjected to routine checking for mycoplasma contamination every two months. Antibody against cleaved-caspase 3 (#9664), PUMA (#98672), p53 (#2527), Akt (#4691), p-Akt (#4060), Foxo3a (#12829), p-Foxo3a Ser253 (#9466), and β-actin (#3700) were obtained from Cell Signaling Technology, Inc. (Beverly, MA). Antibody against Ki67 (ab16667) was obtained from Abcam (Cambridge, UK). The natural compound Tanshinone IIA was purchased from Selleck Chemicals (Houston, TX). Necrostatin-1, z-VAD-fmk, 3-methyladenine, chloroquine; Cisplatin, and 5-fluorouracil were purchased from MedChemExpress (New Jersey, US).

*MTS assay*. MTS assay was performed according to the standard protocol^12^. The cultured cells were resuspended and seeded in 96-well plates at a density of 8x10^3^ cells/well and were incubated at different time points. Cell viability was analyzed with MTS using the Cell Titer 96^®^ Aqueous One Solution kit (Promega Corporation).

*Soft Agar Assay.* The soft agar assay was performed as described previously^13^. Briefly, the agar base was made with 3 mL of Eagle's basal medium supplemented with 0.6% agar and 10% FBS in a 6-well plate. Cells were collected and counted at 8x10^3^ cells/mL concentration in 1 mL of Eagle's basal medium supplemented with 0.3% agar and 10% FBS overlaid into a 6-well plate with 0.6% agar base. The cells were routinely cultured for 14 days. The colony number was counted with the microscope.

*Plate colony formation assay.* The cultured cells were exposed to Tan IIA (0-5 μM), and were routinely incubated for 2 weeks in a 6-well plate (500 cells/well). When cells formed sufficiently large colonies, cells were fixed with 4% paraformaldehyde for 20 min at 37 ˚C. Cells were stained with 0.5% crystal violet for 5 min at 37 ˚C. The number of colonies was counted with a microscope.

*Cell Transfection.* Lentivirus transfection to generate Akt stable knockout cells. In brief, according to the manufacturer's protocol, OSCC cells were transfected with si-PUMA (sc-37153), si-Foxo3a (sc-37887), or siCtrl (sc-37007) purchased from Santa Cruz Biotechnology (Dallas, TX) using the Lipofectamine 2000 (11668019, Thermo Fisher Scientific), and subjected to following assays.

*Immunoblotting.* The immunoblotting (IB) was performed as described previously^14^. Briefly, Cells were lysed in RIPA lysis buffer (Thermo Fisher Scientific, Inc.) containing protease inhibitors to obtain whole-cell extract (WCE), whose concentration was determined by BCA protein assay (Thermo Fisher Scientific, Inc.). Equal amounts of protein (30 μg) were mixed with loading buffer, boiled at 95°C for 5 min, then subjected to SDS-PAGE electrophoresis and transferred onto a PVDF membrane. The membranes were incubated with the primary antibody overnight at 4°C after blocking with 5% non-fat milk at room temperature (RT) for 1 h. Finally, the secondary antibody anti-rabbit/mouse IgG HRP was added and incubated for 30 min at RT, and then the target protein was visualized by chemiluminescence.

*Immunofluorescence (IF).* The IF analysis was performed as described previously^15^. Briefly, CAL-27 cells were fixed in 4% paraformaldehyde (sc-281692; Santa Cruz Biotechnology, Inc.) for 10 min, and permeabilized in 0.2% Triton X-100 (13444259; Thermo Fisher Scientific, Inc.) for 20 min. The slides were incubated with cleaved-caspase 3 antibody overnight at 4°C in a humidified chamber after blocking with 50% goat serum albumin. The next day, the fluorescence-labeled second antibody was added for 40 min at RT. DAPI was used for counterstaining. The stained cell images were obtained using the fluorescence microscope.

*Immunohistochemical (IHC) Staining.* The IHC staining was performed as described previously^16^. Briefly, the tissue slides were dewaxed by immersion in xylene and rehydrated with gradient ethanol. The slides were immersed into boiling sodium citrate buffer (10 mM, pH 6.0) for antigen retrieval, followed by incubation with 3% H_2_O_2_ in methanol for 10 min to block endogenous peroxidase. After blocking with 50% goat serum albumin for 1h at RT, the slides were incubated with primary antibodies overnight at 4°C and then hybridized with secondary antibodies. Finally, the DAB Substrate kit (cat. no. 34002; Thermo Fisher Scientific, Inc.) was used to visualize the target proteins.

*Xenograft mouse model.* All in vivo animal experiments were approved by the Institutional Animal Care and Use Committee (IACUC) of Central South University (Changsha, China). CAL27 cells (2 × 10^6^) in 200 μl DMEM were harvested and subcutaneously inoculated in the right flank of 6-week-old athymic nude mice (n = 6) to generate xenograft models. The tumor volume and body weight of mice were recorded every 2 days. The tumor-bearing mice were randomly divided into two groups when the tumor reached ~100 mm^3^. The compound-treated group was administrated Tanshinone IIA (low-dose group: 10 mg/kg every 2 days, high-dose group: 30 mg/kg every 2 days) by intraperitoneal injection, whereas the control group was administrated the vehicle control. Tumor volume was calculated as length × width^2^ × 0.5. The mice were euthanized at the endpoint, and the tumor tissues were dissected for IHC staining.

*Statistical analysis*. The results were analyzed using SPSS software (version 13.0; SPSS, Inc.) and presented as the mean ± SD. Significant differences between tested groups were analyzed by the Student's t-test or ANOVA. P<0.05 was considered as the criterion for statistically significant.

## Results

### Tan IIA inhibits the cell viability of OSCC cells

**Figure 1:**
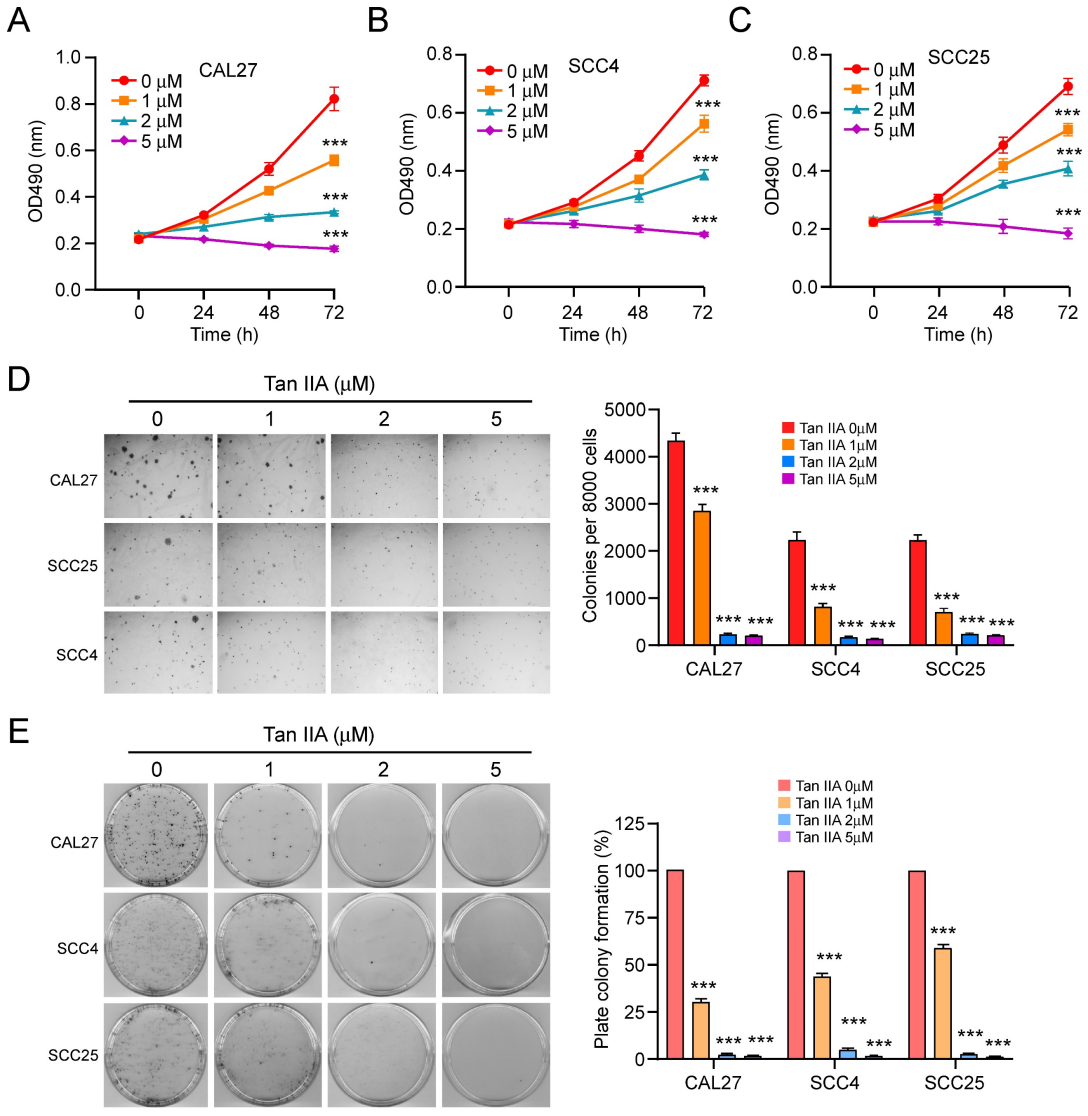
Tan IIA suppressed cell viability and colony formation. (A-C) CAL27 (A), SCC4 (B), and SCC25 (C) cells were treated with different concentrations of Tan IIA (0-5 μM) for indicated times. Cell viability was measured by MTS assay. (D) Colony formation of CAL27, SCC4, and SCC25 cells exposed to different concentrations of Tan IIA (0-5 μM). (e) CAL27, SCC4, and SCC25 cells were treated with Tan IIA (0-5 μM) for 24h, then plated into the 6-well plate, and the plate colony formation was examined. ***, *p* < 0.001.

To determine the inhibitory effect of Tan IIA on OSCC cells, we first detected the cell viability of OSCC cells at indicated time points after different concentrations of Tan IIA treatment (0-5 μM). The MTS data indicated that Tan IIA significantly reduced cell viability dose-dependently in CAL27, SCC4, and SCC25 cells (Figure 1A-C). Furthermore, the colony formation ability of OSCC cells was examined. The results indicated that the colony numbers were markedly suppressed with Tan IIA treatment in CAL27, SCC4, and SCC25 cells (Figure 1D). Consistently, the plate colony formation assay revealed that Tan IIA dose-dependently inhibited the growth of OSCC cells. Exposure to a higher concentration of Tan IIA (2 and 5 μM) exhibited a more potent inhibitory effect on CAL27, SCC4, and SCC25 cells. Especially when the concentration of Tan IIA increased to 5 μM, the colony formation was blocked in tested OSCC cells (Figure 1E). Overall, these results suggest that Tan IIA dose-dependently attenuated the cell viability of OSCC cells.

### Tan IIA induces PUMA-mediated apoptosis in OSCC cells

**Figure 2:**
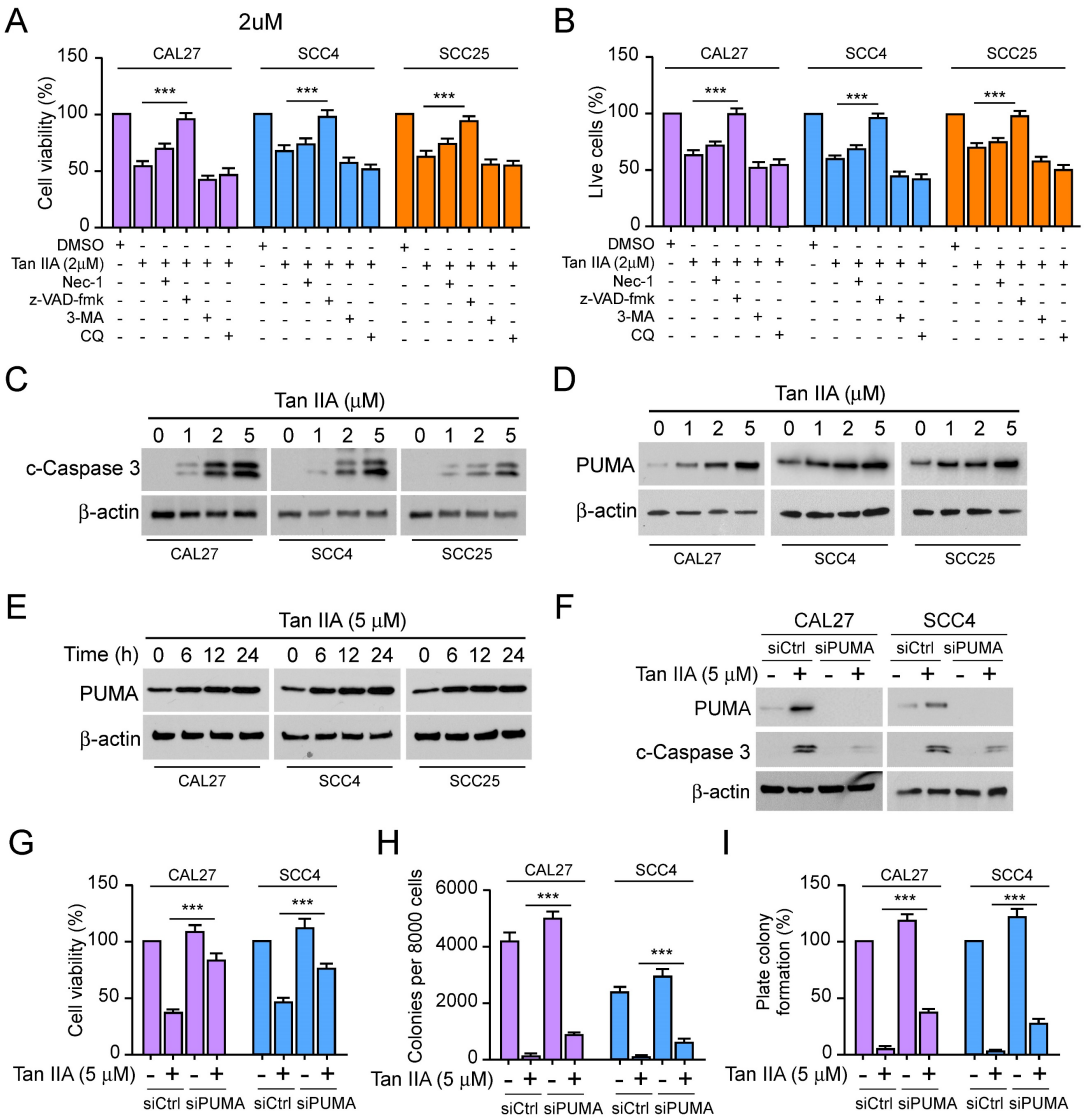
Tan IIA promoted cell apoptosis and PUMA expression. (A and B) CAL27, SCC4, and SCC25 cells were treated with 2 μM Tan IIA combined with Nec-1, z-VAD-fmk, 3-MA, and CQ, respectively. (A) Cell viability was examined by MTS assay. (B) Trypan blue exclusion assay was performed to analyze the live cell population. (C and D) CAL27, SCC4, and SCC25 cells were treated with different concentrations of Tan IIA (0-5 μM) for 24h. The cell lysate was subjected to IB analysis. (E) CAL27, SCC4, and SCC25 cells were treated with 5 μM Tan IIA for indicated times. The cell lysate was subjected to IB analysis. (F-I) CAL27 and SCC4 cells were transfected with siPUMA or siControl for 24 h, followed by 5 μM Tan IIA treatment for 24h. The cell lysate was subjected to IB analysis (F). MTS analysis of cell viability (G). Colony formation ability was measured by soft agar assay (H) and plate colony formation assay (I). ***, *p* < 0.001.

To explore the underlying mechanism that Tan IIA inhibited cell viability and proliferation of OSCC cells. We hypothesized that apoptosis, necroptosis, or autophagy might be implicated in the inhibitory effect of Tan IIA on OSCC cells. As shown in Figure 2A and 2B, the cell viability and live cells were increased moderately in Tan IIA-treated CAL27, SCC4, and SCC25 cells after incubation with RIPK1 kinase inhibitor Nec-1. While treated with Tan IIA and the pan-caspase inhibitor z-VAD-fmk, cell viability and the live cell population were recovered significantly. On the other hand, treatment with Tan IIA and autophagy inhibitors 3-MA and CQ had no significant difference in OSCC cells compared with Tan IIA treatment alone. The results suggested that inhibition of apoptosis but not autophagy rescued Tan IIA-induced inhibitory effects on OSCC cells. The IB data showed that Tan IIA upregulated the protein level of cleaved-caspase 3 and PUMA in a dose-dependent manner (Figure 2C-D). Moreover, the PUMA expression increased time-dependently following exposure to 5 μM Tan IIA (Figure 2E). Conversely, PUMA knockdown prominently suppressed caspase 3 activation (Figure 2F). The cell viability and colony formation ability significantly increased in CAL27 and SCC4 cells (Figure 2G-I), suggesting that PUMA deficiency abolished the inhibitory effects of Tan IIA on OSCC cells. These results indicate that PUMA was required for Tan IIA-induced apoptosis in OSCC cells.

### Tan IIA promotes PUMA via Akt-Foxo3a pathway inhibition

**Figure 3:**
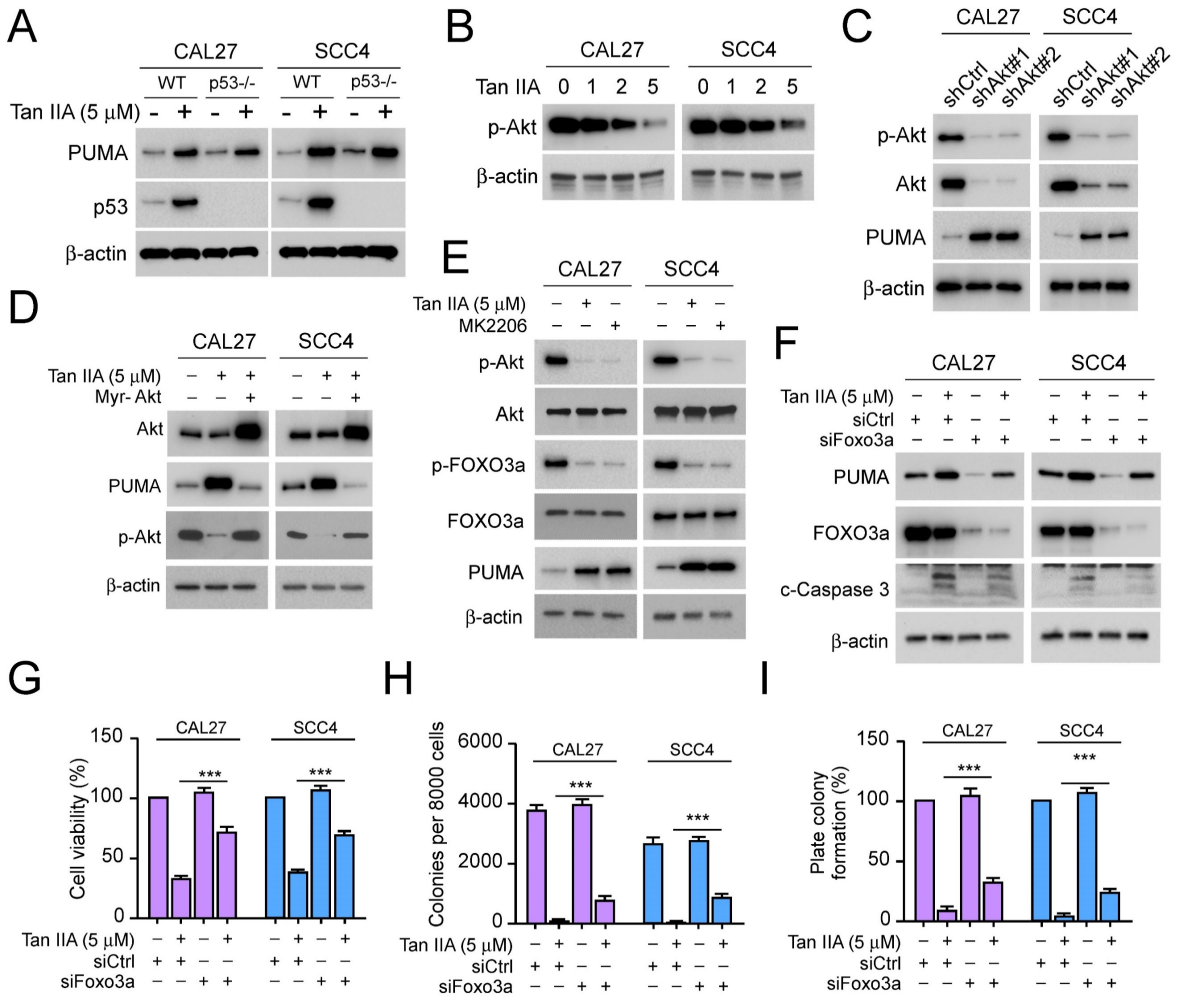
Tan IIA inhibited Akt-Foxo3a signaling. (A) WT or p53**^-/-^** CAL27 and SCC4 cells were treated with 5 μM Tan IIA or not. The cell lysate was subjected to IB analysis. (B) CAL27 and SCC4 cells were treated with Tan IIA (0-5 μM) for 24h. The cell lysate was subjected to IB analysis. (C) IB analysis of CAL27 and SCC4 cells expressing shAkt or shControl. (D) CAL27 and SCC4 cells were transfected with Myr-Akt for 24h, followed by 5 μM Tan IIA treatment for 24h. The cell lysate was subjected to IB analysis. (E) CAL27 and SCC4 cells were treated with 5 μM Tan IIA or MK2206 for 24h. The cell lysate was subjected to IB analysis. (F-I) CAL27 and SCC4 cells were transfected with siFoxo3a or siControl for 24h, followed by 5 μM Tan IIA treatment for 24h. The cell lysate was subjected to IB analysis (F). MTS analysis of cell viability (G). Colony formation ability was measured by soft agar assay (H) and plate colony formation assay (I). ***, *p* < 0.001.

To further determine the role of PUMA for Tan IIA-induced apoptosis, we detected PUMA and p53 expression in CAL27 and SCC4 cells. The IB data showed that Tan IIA markedly enhanced the protein level of PUMA and p53. Intriguingly, with Tan IIA treatment, PUMA expression had no significant decrease in p53**^-/-^** CAL27 and SCC4 cells, suggesting that Tan IIA upregulated PUMA in a p53-independent manner (Figure 3A). And then, we investigated whether Tan IIA exerted an effect on Akt. As shown in Figure 3b, exposure to different concentrations of Tan IIA (0-5 μM), the phosphorylation of Akt decreased dose-dependently (Figure 3B). Meanwhile, Akt knockdown could promote PUMA expression in CAL27 and SCC4 cells (Figure 3C). Conversely, ectopic expression of constitutively activated Akt (Myr-Akt) prominently reduced PUMA and restored Akt phosphorylation in the presence of Tan IIA (Figure 3D). In addition, blockage of Akt activation with Tan IIA and Akt inhibitor (MK2206) treatment overtly suppressed Foxo3a phosphorylation and increased PUMA expression in CAL27 and SCC4 cells (Figure 3E). However, despite exposure to Tan IIA, knockdown of Foxo3a suppressed PUMA and caspase 3 activation (Figure 3F). Consistent with that, the cell viability and colony formation significantly increased in CAL27 and SCC4 cells (Figure 3G-I), which suggested that Foxo3a deficiency compromised the inhibitory effect of Tan IIA on OSCC cells. These results showed that Tan IIA-induced PUMA-mediated apoptosis depended on the Akt-Foxo3a pathway.

### Tan IIA suppresses tumor development of OSCC cells in vivo

**Figure 4:**
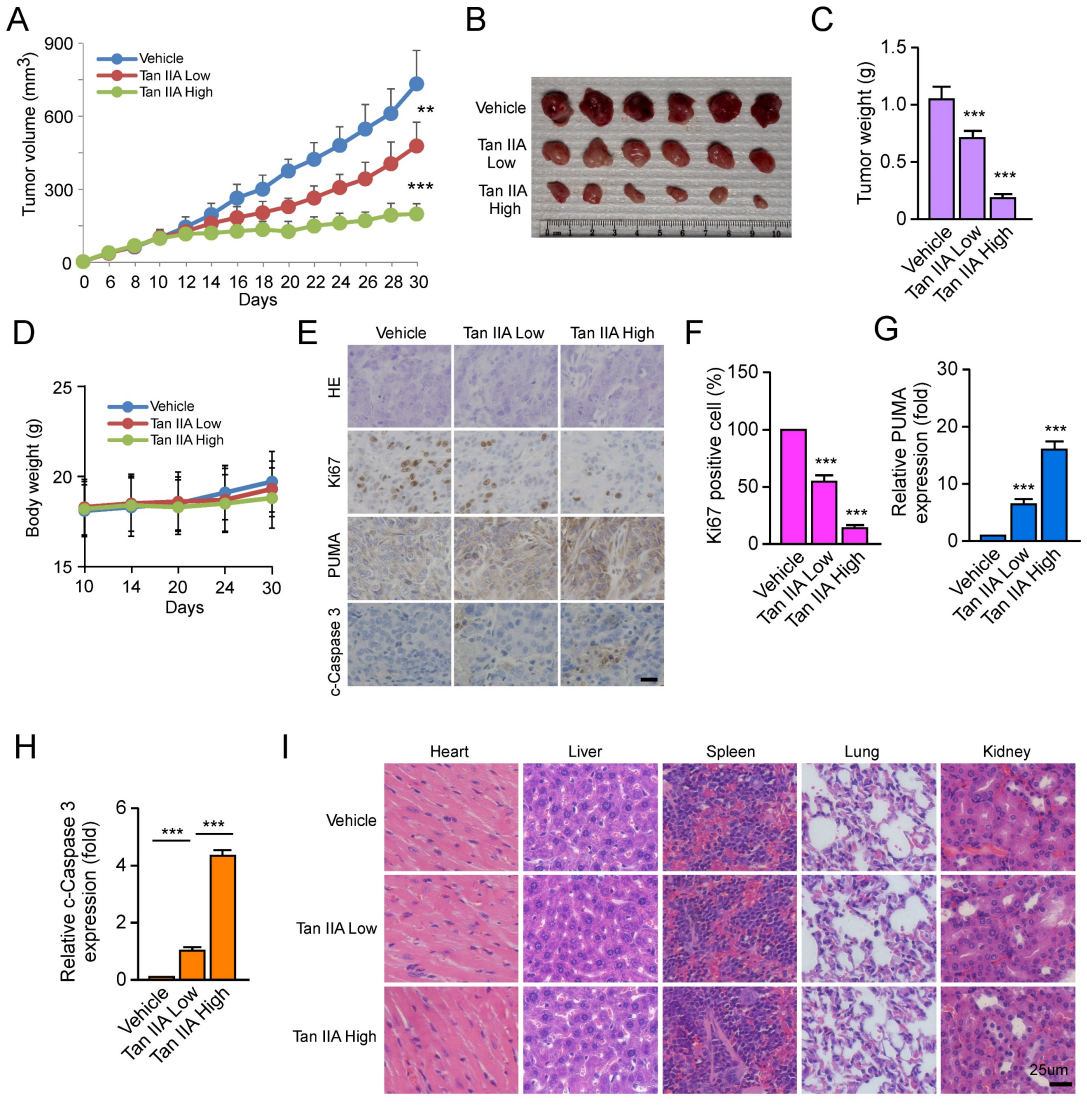
Tan IIA dose-dependently inhibited the tumor growth of OSCC cells *in vivo*. (A-C) The tumor volume (A), The image of tumor mass (B), and tumor weight (C) of CAL27-derived xenograft tumors treated with vehicle, low, and high Tan IIA. (D) The body weight of tumor-bearing mice with the vehicle, low, and high Tan IIA treatment. (E and F) IHC staining of Ki67 and PUMA in CAL27-derived xenograft tumors with the vehicle, low, and high Tan IIA treatment. (G) HE staining analysis of the heart, liver, spleen, lung, and kidney in vehicle- or Tan IIA-treated xenograft tumors. Scale bar, 25 μM, **, *p* < 0.01, ***, *p* < 0.001.

We constructed a xenograft mouse to verify whether Tan IIA inhibited tumor development in vivo. CAL27-deprived xenograft tumors were exposed to low or high dosages of Tan IIA, respectively. The results showed that Tan IIA dose-dependently inhibited the tumor growth of CAL27-deprived xenografts compared with the vehicle control (Figure 4A). The tumor weight of the Tan IIA-treated group was sharply lower than that of the vehicle-treated group in a dose-dependent manner (Figures 4B and 4C). In addition, the body weight did not change significantly between the Tan IIA- and vehicle-treated groups (Figure 4D). IHC staining showed that Tan IIA dose-dependently reduced the population of Ki-67 positive cells and increased PUMA and cleaved-caspase 3 expression (Figure 4E-H). To further examine the toxicity of Tan IIA in vivo, HE analysis showed that Tan IIA administration had no specific cytotoxicity on certain vital organs, such as the heart, liver, spleen, lung, and kidney (Figure 4I). Overall, these results indicated that Tan IIA administration suppressed tumor development of OSCC cells in vivo.

### Tan IIA intensifies the efficacy of CDDP/5-FU-based chemotherapy

**Figure 5:**
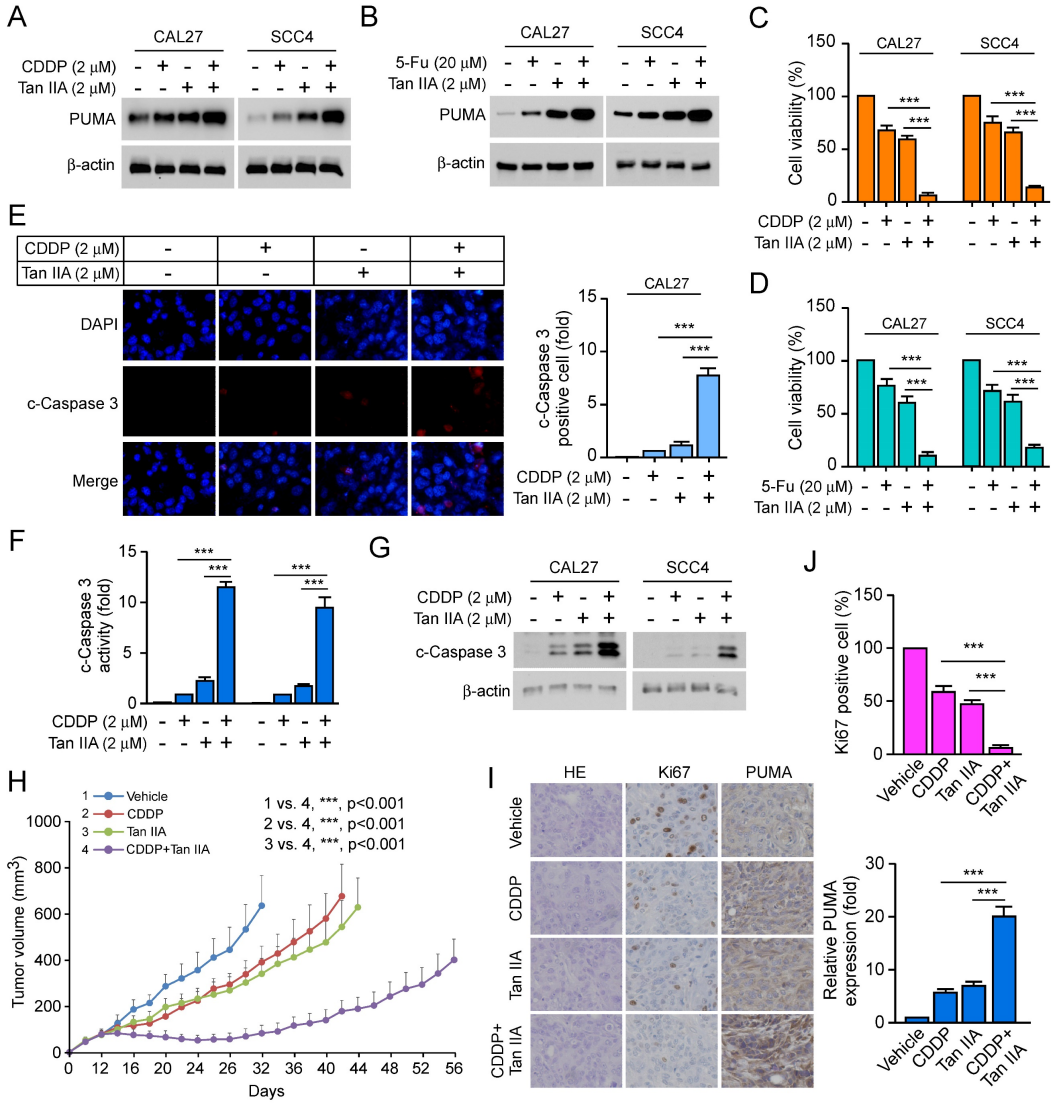
Tan IIA increased the effects of CDDP/5-FU-based chemotherapy by inducing apoptosis. (A and C) CAL27 and SCC4 cells were treated with 2 μM Tan IIA, 2 μM CDDP or combination. The cell lysate was subjected to IB analysis (A). MTS analysis of cell viability (C). (B and D) CAL27 and SCC4 cells were treated with 2 μM Tan IIA, 20 μM 5-FU or combination. The cell lysate was subjected to IB analysis (B). MTS analysis of cell viability (D). (E) CAL27 cells were treated with 2 μM Tan IIA, 2 μM CDDP or combination, and then were subjected to Immunofluorescence (IF) analysis with cleaved-Caspase 3 antibody. (F and G) CAL27 and SCC4 cells were treated with 2 μM Tan IIA, 2 μM CDDP or combination. The cell lysate was subjected to IB analysis. (H) The tumor volume of CAL27-derived xenograft tumors treated with vehicle, Tan IIA, CDDP, or combination. (I and J) IHC staining of Ki67 and PUMA in CAL27-derived xenograft tumors treated with vehicle, Tan IIA, CDDP, or combination. Scale bar, 25 μM, ***, *p* < 0.001.

Cisplatin (CDDP) and 5-fluorouracil (5-FU) were the first-line and broad-spectrum chemotherapy for multiple cancers. We hypothesized that Tan IIA enhanced the efficacy of CDDP/5-FU-based chemotherapy on OSCC cells. The results showed that Tan IIA combined with CDDP increased the expression of PUMA and significantly suppressed cell viability compared with that of Tan IIA or CDDP treatment alone in CAL27 and SCC4 cells (Figure 5A and 5C). Consistently, a combination of Tan IIA and 5-FU exerted a similar effect on CAL27 and SCC4 cells (Figure 5B and 5D). Furthermore, Tan IIA combined CDDP prominently enhanced caspase 3 activity to induce apoptosis (Figure 5E-G). In addition, treatment with Tan IIA plus CDDP inhibited the growth of CAL27-deprived xenografts compared with that of Tan IIA or CDDP treatment alone (Figure 5H). Likewise, IHC staining revealed that Tan IIA combined with CDDP significantly reduced the population of Ki-67 positive cells and increased the protein level of PUMA (Figure 5I-J). Overall, these results suggest that Tan IIA intensified the efficacy of CDDP/5-FU-based chemotherapy on OSCC cells.

## Discussion

Oral cancer is one of the most common causes of cancer-related deaths worldwide^17^-^19^. As the most prevalent type of oral malignancy^20^, ^21^, about 50% of oral carcinoma cases are at advanced stages^22^-^24^. Apoptosis functions as a programmed cell death mechanism, effectively eliminating cancer cells to protect against cancer development^25^, ^26^. However, apoptosis dysregulation is a hallmark of cancer and contributes to tumorigenesis and drug resistance^27^-^29^. PUMA (p53-upregulated modulator of apoptosis) belongs to the Bcl-2 family and induces apoptosis in several cancer cells^30^, ^31^. Excepting that p53 transcriptionally induces PUMA activation, Forkhead box O (FOXO) family member Foxo3a mediates PUMA induction^32^, ^33^. PUMA activation induces apoptosis in either a p53-dependent or -independent manner. Once expressed, PUMA interacts with anti-apoptotic proteins of the Bcl-2 family and directly activates the pro-apoptotic effectors Bax/Bak, leading to mitochondrial outer membrane permeabilization (MOMP), caspase cascades and cell apoptosis in various cancer cells^31^, ^34^, ^35^.

Tanshinone IIA (Tan IIA) is a natural compound extracted from Danshen^8^. Recently, Tan IIA has exhibited a wide range of potent antitumor efficacy against various cancers^2^, ^36^. Tan IIA suppresses various tumors, including leukemia^37^, gastric cancer^38^, non-small cell lung cancer^39^, colorectal cancer^40^, prostate cancer^41^, and hepatocellular carcinoma^42^. The mechanism study revealed that Tan IIA administration induced cell cycle arrest and apoptosis to suppress angiogenesis and metastasis and enhance the antitumor efficacy of chemotherapies 2, 42-44. In our study, Tan IIA exerted a potent inhibitory effect on OSCC cells. Tan IIA dose-dependently attenuated cell viability and proliferation of OSCC cells (Figure 1A-E). Moreover, Qiu et al.^6^ revealed that Tan IIA treatment induced cell apoptosis and upregulated the expression of cleaved-caspase 3. Simultaneously, autophagy was initiated by Tan IIA. From this perspective, we inquired whether Tan IIA has a similar effect on CAL27, SCC4, and SCC25 cells. Intriguingly, under Tan IIA treatment with apoptosis inhibitors z-VAD-fmk, the cell viability and live cells increased to some extent in CAL27, SCC4, and SCC25 cells. In contrast, treatment with Tan IIA and autophagy inhibitors 3-MA and CQ had no significant difference in OSCC cells compared with Tan IIA treatment alone (Figure 2A-B). The results suggested that Tan IIA mediated apoptosis, not autophagy, to execute inhibitory effects on OSCC cells. Further study revealed that Tan IIA upregulated the protein level of cleaved-caspase 3 and PUMA in a dose and time-dependent manner (Figure 2C-E). Conversely, PUMA knockdown prominently suppressed caspase 3 activation (Figure 2F). In addition, PUMA is the downstream target of transcription factor Foxo3a^32^, ^45^. PI3K-mediated Akt activation leads to Foxo3a phosphorylation and cytoplasmic retention, thus preventing PUMA upregulation^46^, ^47^. We manifested that blockage of Akt activation sharply suppressed Foxo3a phosphorylation and increased PUMA expression in CAL27 and SCC4 cells. Our results substantiated that Tan IIA-induced apoptosis and PUMA upregulation leaned on the Akt-Foxo3a pathway. Moreover, MYC and PI3K-Akt signaling synergistically repressed Foxo3a-dependent PUMA expression^48^. It remains unknown whether Tan IIA exerts inhibitory effects on MYC or other apoptosis-associated proteins.

Natural products have been widely studied as vital therapeutic antitumor agents due to their limited toxicity^49^. In our study, Tan IIA dose-dependently inhibited the tumor growth of CAL27-deprived xenografts and had no apparent cytotoxic effect on vital organs. Meanwhile, chemoresistance is one of the major causes of treatment failure in OSCC^50^, ^51^. Cisplatin (CDDP) and 5-fluorouracil (5-FU) are the first-line treatment for advanced OSCC patients. Unfortunately, over 30% of OSCC patients are intrinsically insensitive to CDDP/5FU-based chemotherapy^4^, ^50^, ^52^. However, Tan IIA combined with CDDP/5-FU significantly upregulated the level of PUMA and suppressed cell viability.

Collectively, our study manifested that Tan IIA promoted PUMA-induced apoptosis to exhibit a potent inhibitory effect on OSCC cells and to intensify the efficacy of CDDP/5-FU-based chemotherapy. This evidence indicated that Tan IIA might be a potential therapeutic agent for OSCC treatment.

## Supplementary Material

Supplementary figures.

## Abbreviations

Tan IIA: tanshinone IIA
Nec-1: necrostatin-1
z-VAD: z-VAD-fmk
3-MA: 3-methyladenine
CQ: chloroquine
CDDP: cisplatin
5-FU: 5-fluorouracil
WT: wild type
Foxo3a: forkhead box O3a
Akt: protein kinase B
PUMA: p53-upregulated modulator of apoptosis
PI3K: phosphoinositide 3-kinase
MOMP: mitochondrial outer membrane permeabilization
HK2: hexokinase 2
IB: immunoblotting
IHC: immunohistochemistry
IF: immunofluorescence
