# Supplementary figures and images for: Tanshinone IIA inhibits cell viability and promotes PUMA-mediated apoptosis of oral squamous cell carcinoma
Source: J Cancer. 2023 Aug 6;14(13):2481–90. doi: 10.7150/jca.84537 (PMC10475368; doi:10.7150/jca.84537)

# Supplementary Figure 1

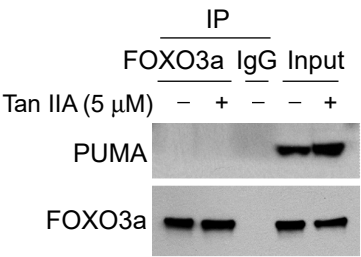

Full gel for Figure 2

C

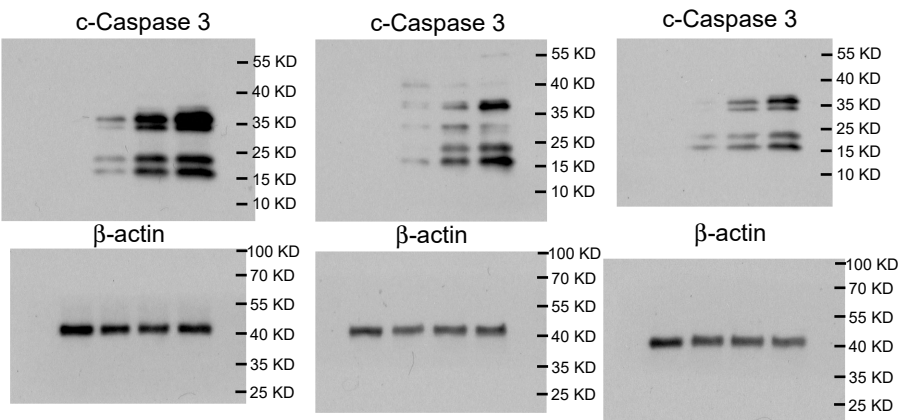

D

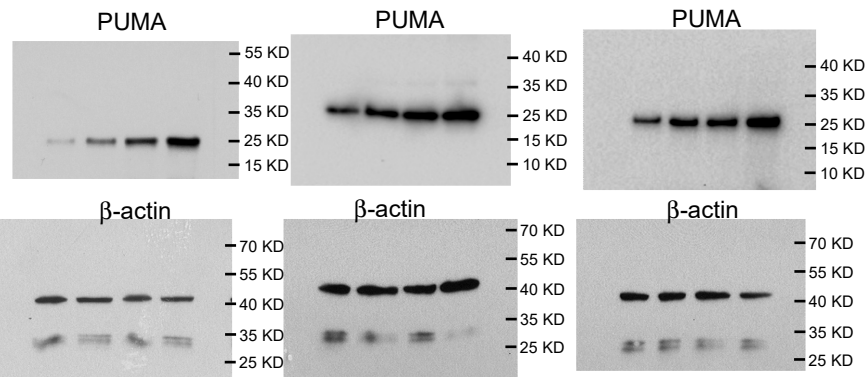

E

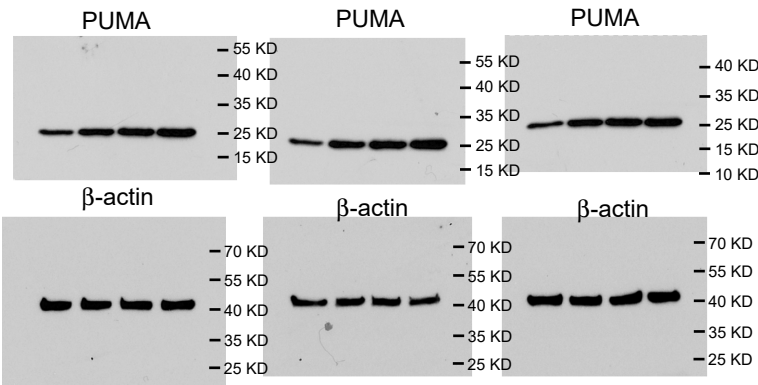

F

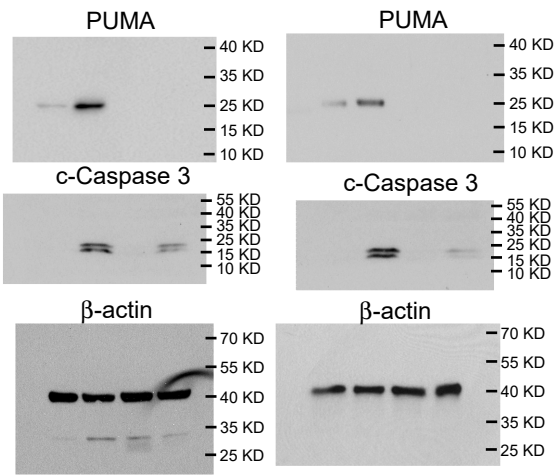

Full gel for Figure 3

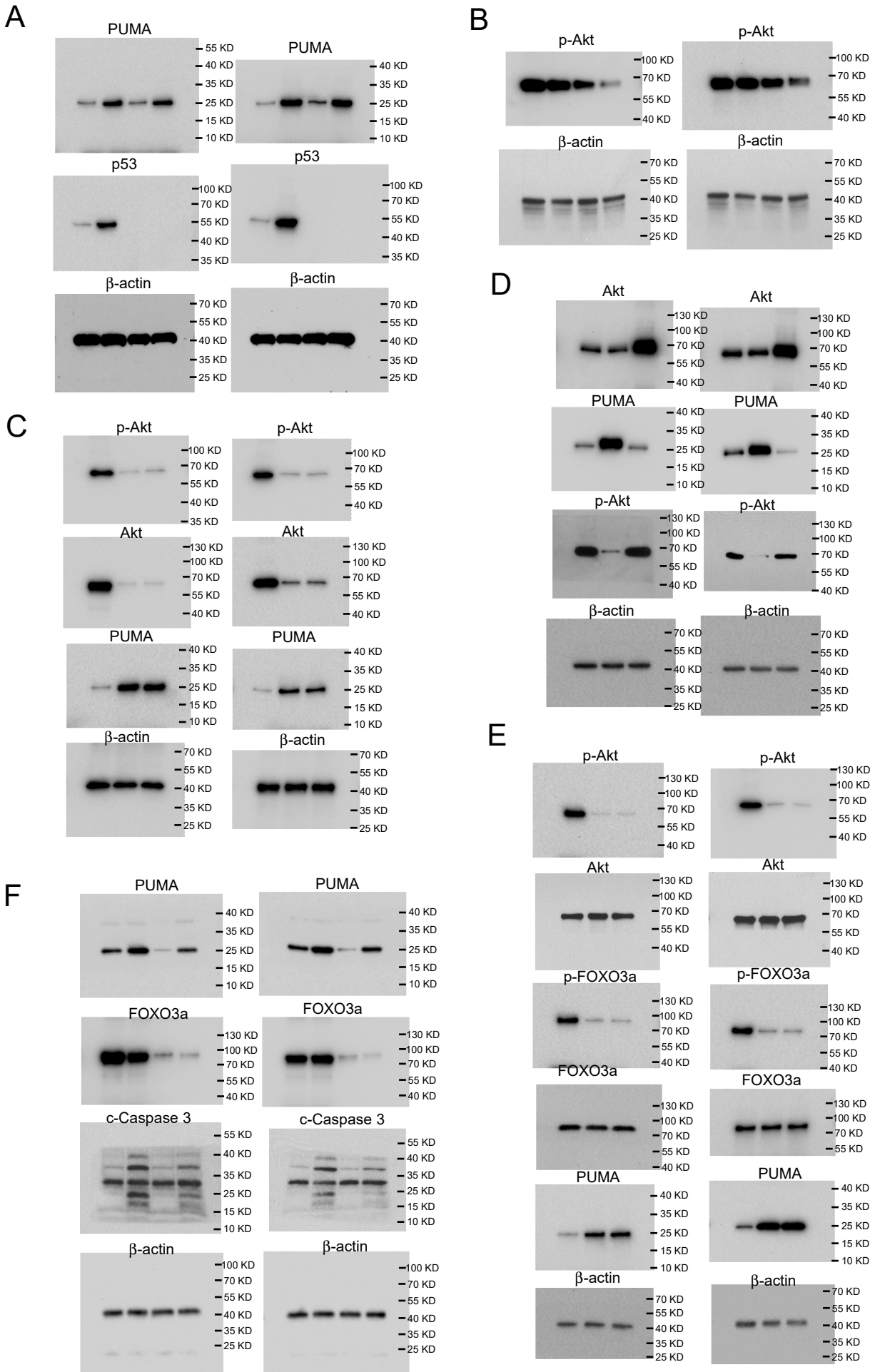

Full gel for Figure 5

A

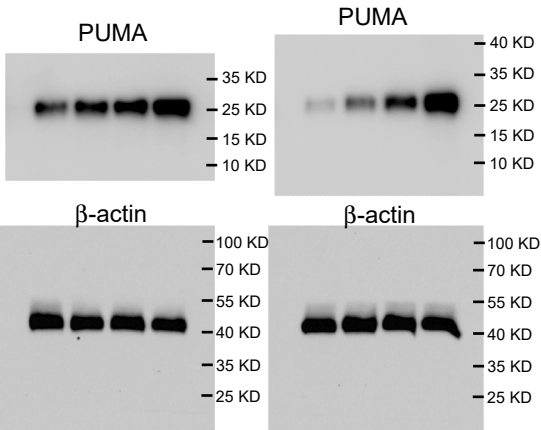

B

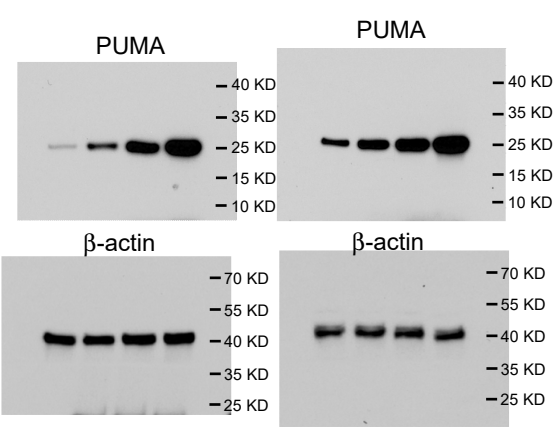

G

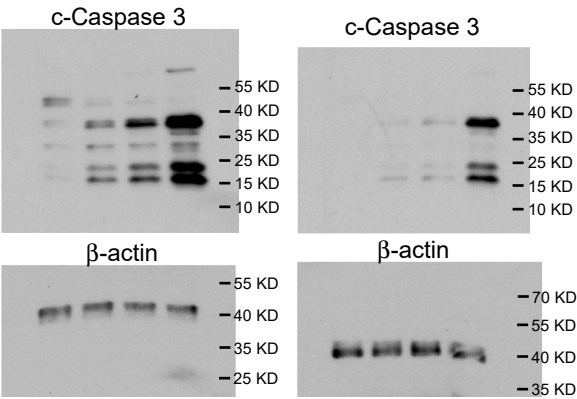

Supplement: Supplementary file 1 — Supplementary figures. [file jcav14p2481s1.pdf]
